# The Transactions of the American Medical Association

**Published:** 1848-12

**Authors:** 


ART. IV.—*The Transactions of the American Medical Association.* Instituted, 1847. Vol. 1: Philadelphia: Printed for the Association. By T. R. and P. G. COLLINS. 1848.

The publication of the transactions of the American Medical Association, which, for some time, has been impatiently looked for, has been at length completed, making a handsome octavo of four hundred pages. The minutes of the annual meeting at Baltimore, May 1848, come first in order, and occupy 46 pages, a condensed abstract of which was submitted to our readers shortly after the meeting was held. The remainder of the volume is devoted to reports of the several committees appointed at the previous annual meeting of the Association, and appended documents.

We had intended, before the transactions were received, to prepare, upon their reception, a digest of these reports for the pages of our journal.

But we find, or their perusal, that the subjects of which treat are as much condensed, in the reports themselves, as due regard for their merits will admit of, and, hence, it would be difficult to do the subjects, or reports, adequate justice without transferring the latter, entire, to our columns. Besides, it is to be hoped that a large portion of our readers will procure copies of the Transactions. We would urge upon all the propriety of so doing, not only because they are well deserving perusal, but in order to encourage and promote the objects of the Association in their publication.

We shall simply enumerate the several reports, and papers annexed thereto, with a few words of comment, and occasional quotations. Some portions we shall select for the electic department of this number, and succeeding numbers of our Journal.

*Report of the Committee on Medical Sciences.*—This report is submitted by Dr. W. T. Wragg, as Chairman of Committee. Prof. Dickson, of New-York, was originally the Chairman of this Committee, but declined serving, owing to pressure of other duties. This report embraces, about fifty pages. The materials, for the most part, are gleaned from contributions to various medical Journals during the previous year. The subjects are classified under the following heads:—Anatomy and Physiology, Hygiene, General Pathology and Therapeutics, and Medical Jurisprudence. The plan pursued by the committee is to select from among the mass of facts, theories, etc., propounded by different writers, during the year, those which seem most worthy of attention. The range of inquiry is extensive, and, as the committee remark, the difficulty is rather in "choosing well, than in collecting abundantly." We could have desired to find some of the topics which are presented, treated at greater length, but this, we are aware, could not be done without rendering the report very voluminous.

The committee express a decided opinion that the fevers distinguished as typhoid and typhus, are identical. In this opinion, as it seems to us, all who have devoted to the subject unbiassed investigation, must concur. We know of no reason why this is not now to be considered one of the *fixed facts* of medical science, except that it would be agreeable to oblige some truly distinguished observers who are committed to an opposite doctrine. We trust, however, and believe, that those observers will not ask this mnch of the complaisance of their brethren, but will be ready, in view of the evidence now before the profession, to confess, frankly, that they have been mistaken. To discover truth is, generally, considered meritorious in proportion to the difficulty attending the discovery; to recede from error is often more difficult than to develop new truth, and, hence, is scarcely less honorable. He is but poorly qualified to make progress in scientific knowledge who resolves that his opinions at all stages of his career, shall be consistent with each other.

*Report of the Committee on Practical Medicine.*—Prof. Joseph M. Smith, of New York, was the chairman of this committee. The report, which covers above thirty pages, is wholly occupied with Epidemic diseases, and, chiefly, with Epidemic Typhus. A general account is given of the fatality of this disease in different parts of the country, and of its supposed causes, which is somewhat meagre in its character, although, it is to be presumed, the author appropriated all accessible information on the subject. It is deeply to be regretted that we have not more complete historical accounts of this disease as it has been presented at different places, and, particularly, at New York city, where the cases have been so numerous. Several papers of interest and value have been communicated, but what we could desire, is, not only the deductions or opinions of those who have had opportunities to study the disease, but data from which members of the profession may be able to draw, each his own conclusions. We could wish, in other words, for such histories as the history of Continued fever at the Hospital La Charite, by Louis—histories, in the first place, embracing a large collection of cases, and, in the second place, based upon observations of their symptoms, treatment, pathological appearances, etc., accurately observed, carefully recorded and numerically collated. The character of the fever, in the living and dead body, would then have been determinable; we should not have to make allowances for the bias of pre-conceived opinions, and the results would be as reliable, and perhaps as valuable, a century to come, as now. We had hoped for such a history of the epidemic at New York from the committee appointed by the Academy of Medicine, but we have looked in vain for the final report which was promised in the report of their preliminary examinations. A tempting opportunity for meritorious services and distinction has been suffered to pass by unimproved, to say nothing of (what is of much greater consequence) the loss to science and humanity. Some valuable contributions to numerical investigations of this disease have been made, but their value has been limited in consequence of their being restricted to a small number of cases. The report of Dr. Sargeant, of Philadelphia, deserves particular commendation in this connection.

Prof. Smith considers at some length, the identity of Typhoid and Typhus fevers, and arrives at the conclusion that they are identical.

Appended to this report is a truly valuable paper by Gurdon Buck, M.

D., Surgeon to the New York Hospital, etc., on "*Oedematous Laryngitis, successfully treated by scarification of the glottis and epiglottis.*" The details of five cases are given in which scarifications were practiced with success in this imminently dangerous form of disease. The subject is illustrated by four well executed engravings. This paper will claim farther notice hereafter.

*Report of the Committee on Surgery.*—This report is by Dr. Geo. N. Norris, and, as the reader would expect, after naming the author, it is an excellent digest of contributions to the stock of surgical knowledge during the previous year. Considerable space is, of course, allotted to anæsthetic agents. Accompanying the report, is an essay on the latter subject by H. J. Bigelow, M. D., of Boston, Mass., together with statistics derived from the Mass. General Hospital, and communications from several surgeons in relation to the same subject. The committee also communicated a paper by Dr. A. Dubois, on "*Opthalmitis Post Febrilis,*" *a severe form of inflammation of the eye, following Typhus fever, as it appeared in the city of New York in* 1847-8.

This report extends over more than sixty pages of the volume.

*Report of the Committee on Obstetrics.*—This is brief, occupying only seven pages. Dr. Harvey Lindsly was the chairman of this Committee. The application of anæsthetic agents to midwifery practice is the only subject discussed. Appended to the Report, are the opinions of several eminent obstetricians, respecting the safety and advantage of anæsthetic influences in labor; also a communication from Dr. Markall, of Maryland, on the use of ice to promote uterine contractions; and a communication by Dr. Blackburn, on the use of a decoction of the roots of the cotton plant, as an emmenagogue.

*Report of Committee on Education.*—Prof. A. H. Stevens was Chairman of this committee. After some sound and judicious remarks upon the present state of Medical Education in the United States, the circumstances which affect it unfavorably, and the means by which improvement is to be made, the committee submitted several resolutions, which we quote in full, as amended and adopted by the Association. They are interesting and valuable as expressive of the sense of the majority of the members of the Association with respect to Medical Education—a subject of vast importance, and which, more than any other subject, was involved, in the origin of the Association. The Resolutions are as follows:—

1. *Resolved, therefore,* That this Association earnestly and respectfully appeal to the trustees of hospitals to open their wards for the purposes of clinical instruction, satisfied that they will thereby more efficiently aid the cause of humanity, and more perfectly accomplish the benevolent intentions of the founders of the charity.

2. *Resolved*, That this Association considers defective and erroneous, every system of Medical instruction which does not rest on the basis of practical demonstration and clinical teaching, and that it is therefore the duty of the medical schools to resort to every honorable means to obtain access for their students to the wards of a well-regulated hospital.

3. *Resolved*, That the practice of appointing physicians and surgeons to the charge of an hospital on political or other grounds than those of professional and moral worth, is inconsistent with the welfare of its inmates, and, of consequence, inhumane and unjust, subversive of the objects of its founders, and incompatible with a conscientious association of the high responsibilities devolved on the appointing power.

4. *Resolved*, That this committee reiterate and strongly recommend to the Association a practical observance of the resolutions appended to the report of the committees on preliminary education, and on the requisites for graduation submitted to the Medical Convention which assembled in Philadelphia in May, 1847.

5. *Resolved*, That the faculties of the different schools be requested and advised to institute daily or weekly examinations recapitulatory of the previous lecture or lectures, and take such measures as may enable them to ascertain the regular attendance of the students upon the lectures up to the close of the term.

6. *Resolved*, That this Association recommend to the faculty of each medical school to conduct the final examination of candidates for the diploma, in presence of some official person or persons properly qualified to recognize the attainments of the candidate, but who has no pecuniary interest in the institution, or in the number of its pupils.

7. *Resolved*, That it be also recommended, that in lieu of the usual inaugural thesis, or in addition thereto, each candidate for the diploma be required to present to the faculty at or before the time of final examination, a report drawn by himself, and from his personal observation of not fewer than five cases of disease, and upon which he shall be duly examined.

The difficulty of obtaining information upon some of the subjects upon which it is required to report, induces your committee to offer also the following:—

8. *Resolved*, That the faculty of each medical school be requested annually, and as early as possible to furnish the chairman of the committe on education, with a statement of the number of pupils and graduates of in their respective schools, together with such other information as may expedite the labors of the committee, and enable it to discharge the duties assigned by the constitution under which it acts.

*Report of the Committee on Medical Literature.*—This report, by the chairman of the Committee, Prof. O. W. Holmes, has much of that vivacity and pungency which characterize the author's writings. It has been thought by some to contain some remarks a little too caustic in their tone; but this arises, doubtless, from the author's epigrammatic style, which leads him to express, occasionally, more than, probably, he intends. We may say of this, as of the other reports, that, to have done the subject full justice, it should have been more elaborate, but, then, it should have been greatly extended, and would be less likely to have been generally perused. The author shows, as we think, a spirit of fairness and candor, and his strictures appear to us, in the main, just. It is to be considered that the duty of this committee was peculiarly difficult and delicate. It is far easier to gather together important facts, for a given period, bearing upon a particular department of science, than to examine all that comes under the denomination of medical literature for the same period; and to report upon the relative merits of the latter, is not a very agreeable office, when it is considered that the contributor of every article for a medical Journal thinks his effusion should by no means be overlooked. Dr. H., after giving a brief historical sketch of the rise and progress of medical journalism in the United States, notices, seriatim, all the medical Journals now published in this country, mentioning some of the more interesting articles which they have, respectively, contained. That some valuable articles have been overlooked in this synopsis is certainly true, but it could hardly be otherwise.

On behalf of the craft, we might take exceptions to his comments on the repetition of similar articles in different periodicals. To quote his language, "the ring of editors sit in each other's laps with perfect propriety, and great convenience it is true, but with a wonderful saving in the article of furniture." We look upon this as a *jeau d'esprit*, at which we can laugh as heartily as any other reader, and as at any other good natured hit. In this light we presume the author considers it, for he must, of course, be sensible that one of the functions of a journal is to reproduce for its readers the valuable articles which appear in contemporaneous periodicals. This would be superfluous if each journal circulated among the same readers, but it is otherwise; each has its own circle. If any injustice were done to the journals whose pages furnish the most liberal stock for general appropriation, *they* would be likely to complain, but who ever heard of such a complaint! We have heard complaints that some journals did not do justice to others in its selections, but never that they copied too liberally. So far from being considered a disadvantage, it is quite the other way, every excellent article which creeps into other journals being so much testimony to the value of the journal from which it is taken. The real subjects of the satire are members of the profession generally, who do not contribute to medical periodicals as liberally as they should; and, in this respect, the satire hits the author of the report more than any other individual that we know of. Why does he not write oftener for medical journals himself, and assist to save us poor editors from sitting so much in each other's laps? We promise that our Journal shall contain as many number of pages of eclectic matter less the usual average, as he will occupy by something from his own pen.

We quote what Dr. H. says of Introductory Lectures:—

"In connection with periodical literature, it seems proper to allude to the 'INTRODUCTORY LECTURES," of which so large a number are delivered and printed annually. They must not be judged too harshly, for they are delivered to young men, who like high seasoning, and they naturally partake somewhat of the character of advertisements. Many of them are agreeable and appropriate performances, but others are open to severe comment. Turgid and extravagant attempts at eloquence, a fondness for effete Latin quotations, a parade of scholastic terms where simple ones only are called for, an inclination to adopt the cant phrases of political and literary writers, are the common faults of these productions. The physician should remember, that his style has no more occasion for pomp of oratory and glitter of epithet, than his costume for the gold lace and feathers which belong to the chieftain. Nothing is more offensive than an attempt to tell that which should be said plainly and decently, in high flown language. It vitiates the taste of the student who listens to it or reads it, and exposes the profession to derision from those who cannot value the important truths disguised by such ill chosen finery."

For the benefit of those who may not peruse the report, we make, further, the following quotations:—

"It cannot be denied that the great *forte* of American Medical Scholarship has hitherto consisted in "*editing*" the works of British authors. The Committee are not disposed to disguise the fact that this business has been carried on in a very cheap and labor-saving fashion. A tacit alliance between writers and publishers has infused the spirit of trade into the very heart of our native literature. The gilt letters of the book-binder play no inconsiderable part in the creation of our literary celebrities. Sometimes the additions by the "American Editor" have been real and important, oftener nominal and insignificant. The following calculation of the proportion added to different recently published works, taken at random, will show the average amount of material so contributed. The Editor's proportion was, in two instances, one-fourth; in two more, one-eighth; in one, one-ninth; in another, one-tenth; in others, one-fifteenth, one-seventeenth, one-nineteenth, one-twentieth, one-twenty-eighth, one-fifty-ninth, one-sixty-fifth, one-ninetieth, one hundred and seventh, and, in one instance, such a sprinkling as a penful of ink might furnish, and leave enough to spare for a flourishing autograph. The fairest fruits of British genius and research are shaken into the lap of the American student and the great danger seems to be, that in place of the genuine culture of our own fields, the creative energy of the country shall manifest itself in generating a race of *curculios* to revel in voracious indolence upon the products of a foreign soil!

In viewing the great branches of Anatomy, Physiology, Surgery, Obstetrics, and Practical Medicine, it will be seen at once that the first four are essentially the same to the American student that they are to his European models. As might be expected in a new country, the practical branches have almost exclusively occupied the attention of the medical student. The contributions to Anatomy and Physiology have been few, and for the most part insignificant compared with those which have emanated from the countries of Hunter, of Bichat, and of the Meckels. Of all the practical branches, Operative Surgery, a most important and attractive pursuit, but still, as its name (chirurgery) literally imports a *handicraft*, has been the favorite, and whatever credit belongs to boldness, ingenuity, and dexterity, may be claimed, without fear of dispute, for its American practitioners.

Two fatal influences have acted not merely on medical science, but on all natural science in this country. The first is the habit of indolence generated by the easy acquisition of a foreign literature which seems to answer every necessary purpose. The second is the habit of negligence which springs from the curious fact of a constant parallelism, which is not identity, in most natural objects and phenomena of the New World, with something of the older continent. In literature this has enfeebled the relation between words and realities; in science it has induced the same laxity and incoherence. The American constitution must be studied by itself—it differs from the European in outline, in proportions, in the obvious characters of the skin and hair—why should it not differ in the susceptibilities which, awakened, become disease? The American climate re-moulds the European, and casts a new die of humanity—will it not generate cause of disease different from those of the Old World. Over this virgin soil a new Flora is weaving her long web of tapestry, flowing from the lichens of Katahdin to the myrtles of Cape Sable; is there no undiscovered healing in any of its leafy and blossoming folds? Here is the true field for the American medical intellect; not to set English portraits of disease in American frames; not to trust for immortality to a little more or less of manual adroitness or questionable hardihood; but to co-operate with that fast-gathering band of students who, in other departments of science, are studying what nature has done with her American elements, and teach us what disease is here, how it is generated, and what kindly antidotes have been sown in the same furrows with its fatal seeds."

Passing by the reports of the chairman of the committee on Publication, and the treasurer, Dr. Isaac Hays, which are occupied with financial details, we come, next, to a code of ethics adopted by the Philadelphia college of Pharmacy, and reported to the Association by the officers of the college. The code prohibits members of the college from originating and preparing medicines, the composition of which is concealed from other members, or from regular physicians, and *recommends* "the propriety of discouraging their employment when called upon for an opinion as to their merits." This is very well as far as it goes, but rather a moderate step in the way of reform. We take pride in stating that the two apothecary shops in this city to which the greater portion of the prescriptions of the physicians of the city are carried, have discontinued the sale of secret remedies. Good resolutions are very well, but good acts are far better. We would say to the members of the Philadelphia College of Pharmacy, do as the two most prominent apothecaries of Buffalo have done.

Next follows a *statement in relation to the United States Naval corps*, by two delegates to the Association from the U. S. Navy. It appears from this report, that the Naval corps, "after long continued and arduous efforts," have attained an "assimilated rank with medium classes of their military brethren." The Association passed a resolution expressing their gratification at this fact, which was communicated to the Hon. the Secretary of the Navy, by whom its reception was subsequently acknowledged in complimentary terms.

Next, follows a *"communication on Hygiene, from the medical department of the National Institute."* This communication, after some considerations tending forcibly to portray the importance of investigation with reference to sanitary improvements, closes with the following recommendations:—1st, The establishment of a permanent committee on Hygiene. 2d, A recommendation to the various State Legislatures to establish throughout the Union, uniform systems for the registration of births, deaths and Marriages." On reference to the proceedings of the Association, we perceive, that, after being referred to a committee for consideration, the above recommendations were adopted, and a committee of twelve appointed, of which, James Wynne, M. D., author of the communication from the Institute, was made chairman.

Thirty pages of the transactions are devoted to a report of T. O Edwards, M. D., of Ohio, member of Congress, being the substance of a report made to the House of Representatives on the importation of spurious drugs. This able report has been pretty extensively circulated as a public document, and has been already noticed in our pages. We are glad to see it among the transactions of the Association.

The standing Committee on *Registration of Births, Marriages, and Deaths*, by the chairman, Dr. Jno. H. Griscom, made a short report, stating that a law providing for such registrations in the state of New York, had gone into operation, and that steps have been taken in several states, preliminary to carrying into effect the recommendation of the Association on this highly important subject.

*Report of the Committee on Indigenous Medical Botany.*—This report is quite brief (seventeen pages) considering the wide range of investigation which the subject admits of, and indeed claims. The chairman, Dr. N. S. Davis, after some remarks on the importance of the study of Botany to the medical practitioner, notices the following indigenous plants:—Rumex, Yellow Dock, Hamamelis, Witch Hazel; and Cimicifuga Racemosa, Black Cohosh. The author states, by way of apology for the meagerness and limited extent of the report, that although he addressed letters to all the members of the committee, (which were numerous, embracing practitioners in several states) soliciting materials for the report, he received no returns except from two, and these at too late a date to incorporate in the report. This committee, by a resolution of the Association, were instructed to continue their researches during the present year. Next, follow reports on the number of medical practitioners in the states of Virginia, Massachusetts and Delaware, respectively. Next comes a paper communicated by Gurdon Buck, Jr., M. D., giving an account of "*a new feature in the anatomical structure of the genito-urinary organs, not hitherto described.*" This is illustrated by a plate. We must refer the reader to the paper itself, for an account of this supposed anatomical discovery. Finally, the volume concludes with a catalogue of the officers and permanent members of the American Medical Association.

In closing this skeleton review, we would again express the hope that this volume, and succeeding volumes of the Transactions of the Association, may have an extensive circulation among the Profession of this country. Their intrinsic value, doubtless, is a sufficient basis for this recommendation; but, in addition to this, an interest in the Association, and an American spirit will thereby be fostered. The circulation of these annual transactions will, also, tend, in no small degree, to stimulate the exertions of those appointed to report on scientific subjects; it will encourage voluntary communications; and, what is very essential, will furnish a revenue sufficient for the expenses of publication.

In contemplating what has already been accomplished by the Association, and its present position, the Profession have much cause for gratulation. The difficulties always attendant upon the inception of a great enterprise, have been happily surmounted, and it now only remains to sustain and perpetuate an institution, which promises results equal to the anticipations of the most zealous and enthusiastic of its founders.

